# Remifentanil Patient-Controlled Analgesia for Labor Analgesia at Different Cervical Dilations: A Single Center Retrospective Analysis of 1045 Cases

**DOI:** 10.3390/medicina61040675

**Published:** 2025-04-06

**Authors:** Tatjana Stopar Pintaric, Lara Vehar, Alex T. Sia, Tomislav Mirkovic, Miha Lucovnik

**Affiliations:** 1 Clinical Department of Anaesthesiology and Intensive Therapy, University Medical Centre, 1000 Ljubljana, Slovenia; tomislav.mirkovic@kclj.si; 2 Faculty of Medicine, University of Ljubljana, 1000 Ljubljana, Slovenia; larra.vehar@gmail.com; 3 Department of Women’s Anaesthesia, KK Women’s and Children’s Hospital, Singapore 229899, Singapore; alex.sia.t.h@singhealth.com.sg; 4 Clinical Department of Perinatology, University Medical Centre, 1000 Ljubljana, Slovenia; miha.lucovnik@kclj.si

**Keywords:** labor analgesia, remifentanil-PCA, labor

## Abstract

*Background and Objectives*: Remifentanil is a potent synthetic μ-opioid receptor agonist known for its rapid onset and ultrashort duration of action, making it a popular choice for intravenous labor analgesia. The analgesic effectiveness of remifentanil patient-controlled analgesia (Remifentanil-PCA) may vary based on the stage of labor and parity, potentially influencing satisfaction with labor analgesia. This study aimed to evaluate the degree of pain reduction achieved with remifentanil-PCA, considering different cervical dilations in both nulliparous and multiparous women. *Material and Methods*: Women who were ≥37 weeks pregnant with singleton cephalic fetuses, either by spontaneous onset or induction of labor, were included in the study. Data were collected from the Labor Record form, which included demographic and obstetric information, as well as the onset of analgesia categorized by cervical dilation (1–3 cm, 4–6 cm, 7–9 cm, and full dilation). Additionally, data on analgesia onset and duration (the time interval between the start of analgesia and the delivery of the baby), initial numerical rating scale (NRS_0_) for pain intensity, NRS after the first hour of analgesia (NRS_1_), the lowest recorded NRS during labor (NRSmin), and pain reduction during the first hour of analgesia (NRS_0_–VAS_1_), satisfaction with labor analgesia (rated 0 for dissatisfied, 1 for moderately satisfied, 2 for very satisfied), and complication rates were obtained from the remifentanil-PCA form. *Results*: A total of 513 nulliparas and 523 multiparas who gave birth between 1 January 2019 and 31 December 2019 were reviewed. No significant differences were found between the two groups regarding age, body mass index, labor induction rates, occipito-posterior positioning, blood loss > 500 mL, or neonatal outcomes. Nulliparas exhibited a higher gestational age (*p* = 0.021), longer labor duration (*p* < 0.001), and increased rates of cesarean sections (*p* < 0.001) and vacuum extractions (*p* = 0.002). Remifentanil-PCA consistently provided mild to moderate pain intensity reduction. No differences were found in VAS_0_, VAS_1,_ or pain intensity reduction (VAS_0_–VAS_1_) regardless of the stage of labor or parity. Significant differences in VAS min were observed among nulliparas at different stages of labor (*p* < 0.026). However, a higher proportion of multiparas reported moderate (24.7% vs. 9.5%, *p* < 0.001) and high satisfaction (90% vs. 75%, *p* < 0.001) with remifentanil-PCA compared to nulliparas. Importantly, no serious complications in mothers or neonates attributed to remifentanil-PCA were observed during the observational period. *Conclusions*: Remifentanil-PCA demonstrates consistent effectiveness regardless of the stage of labor or parity. This indicates that remifentanil-PCA can be administered at any point during labor. Coupled with its rapid availability and immediate analgesic effect, this feature enhances the flexibility of its use in clinical practice.

## 1. Introduction

Remifentanil is a potent synthetic μ-opioid receptor agonist known for its rapid onset and ultrashort duration of action, making it a popular choice as an intravenous agent for labor analgesia. It is typically administered via the patient-controlled analgesia (PCA) modality to help women manage labor pain, whose intensity often fluctuates due to uterine contractions. Remifentanil is rapidly metabolized by plasma esterases into inactive metabolites, independent of renal and liver function. With a very short context-sensitive half-time of approximately 3.5 min, remifentanil poses a minimal risk of accumulation in the body. Additionally, it crosses the placenta and is quickly redistributed and metabolized by the neonate, indicating that any potential side effects for both mother and child are likely to be transient [1].

While the pain reduction provided by remifentanil-PCA may vary, prior well-designed studies have shown that it offered reasonable levels of maternal satisfaction compared to neuraxial analgesia, allowing parturients to manage labor pain effectively [2,3,4,5]. This characteristic may be particularly advantageous in rapidly progressing and advanced labors, where the administration of epidural anesthesia (EA) could be technically more challenging due to intense and frequent contractions, and when EA could not be accomplished on time [6,7,8].

However, the experience of labor pain differs among parturients and is influenced by various factors, including parity, duration of labor, rate of progression, pelvic anatomy, fetal size, fetal presentation, and the induction or augmentation of labor [9]. Previous research has highlighted interactions among these factors; for instance, nulliparous women tend to experience more severe pain during the onset of the active phase compared to the other phases of the first stage of labor. In contrast, multiparous women often report the most intense pain during the second stage of labor. These differences may have significant implications for the selection and administration of analgesic therapy [10].

Recently, five quality indicators for obstetric anesthesia have been identified, one of which pertains to the effectiveness of epidural analgesia within 45 min after the initiation of the epidural infusion [11]. However, there are currently no similar standards applied to intravenous remifentanil analgesia during labor. Given its increasing popularity as a pain relief modality in centers like ours, it is crucial to evaluate the quality of remifentanil analgesia more systematically.

This study aimed to assess how pain reduction varied with different stages of labor (measured by cervical dilation) and parities (nulliparous vs. multiparous) among women receiving remifentanil-PCA. Secondary outcomes included satisfaction with labor analgesia, as well as maternal and neonatal side effects.

## 2. Material and Methods

With approval from the Ethics Committee of the Republic of Slovenia (No. 0120-504/2022/3), we conducted this retrospective observational study utilizing the electronic records of women who received remifentanil-PCA for labor pain relief at the Department of Perinatology, University Medical Centre Ljubljana, during the period from 1 January 2019 to 31 December 2019. The study included women with singleton pregnancies at term (≥37 weeks of gestation) with fetuses in cephalic presentation who were admitted to the labor ward following spontaneous onset or induction of labor. Given the retrospective nature of the study, we adhered strictly to the STROBE (Strengthening the Reporting of Observational Studies in Epidemiology) guidelines.

## 3. Data Collection

Data were obtained from the electronic “Labor Record” and “Remifentanil Patient-Controlled Analgesia (PCA)” forms stored in the Unified Document System of the University Medical Centre Ljubljana, Slovenia. From the Labor Record, we collected data on the mother’s age, body mass index (BMI), parity, gestational age, infant weight, occurrence of occipito-posterior position, mode of delivery (cesarean section or vacuum extraction), Apgar score at 5 min (<7), incidence of perinatal asphyxia (defined as a pH in the umbilical artery < 7), admission to the Neonatal Intensive Care Unit (NICU), and the timing of onset of analgesia categorized by cervical dilation (1–3 cm, 4–6 cm, 7–9 cm, and 10 cm or full dilation).

From the remifentanil-PCA form, we obtained data on the initiation and the duration of analgesia (the time interval between the start of analgesia and delivery), initial Visual Analog Scale (VAS_0_) scores for pain intensity (where a score of 0 = no pain and a score of 10 = worst possible pain), VAS after the first hour of analgesia (VAS_1_), the pain reduction during the first hour of analgesia (VAS_0_–VAS_1_), the lowest recorded VAS during labor (VAS_min_), the maximum dose of remifentanil used in the first hour of analgesia, overall satisfaction with labor analgesia (rated as 0 = dissatisfied, 1 = moderately satisfied, 2 = highly satisfied), and the occurrence of respiratory or other complications. We also collected data on any concurrent use of other analgesics and any conversions from remifentanil-PCA to epidural analgesia or vice versa.

All collected data were verified independently by an anesthesiologist (TSP) and an obstetrician (ML). Each parturient received the remifentanil-PCA regimen in accordance with the standard operating protocol of the Department of Anesthesiology and Intensive Therapy at the University Medical Centre Ljubljana. Remifentanil hydrochloride (Ultiva, Glaxo Smith Kline, Oslo, Norway) was diluted in normal saline to a concentration of 40 µg/mL and administered in a dose ranging from 20 to 40 µg (starting with a higher dose for multiparas and for parturients in an advanced stage of labor). The bolus was delivered at a constant flow rate of 1.67 mL/min, with a lockout interval of 2 min and no background infusion (Rhythmic™ Evolution, Micrel Medical Devices, Athens, Greece). Parturients were continuously monitored for oxygen saturation via pulse oximetry (SpO_2_), heart rate, and end-tidal CO_2_ (etCO_2_) using Capnostream^®^ capnography (Oridion^®^, Jerusalem, Israel), with blood pressure checked every 30 min. Each parturient received mandatory one-to-one midwifery care and supplemental oxygen (2 L/min) via a nasal catheter. Cardiotocography (CTG) was continuously monitored using either the Hewlett Packard Viridia Series 50IP^®^ (Hewlett Packard, Palo Alto, CA, USA) or the Philips 50XM^®^ (Amsterdam, The Netherlands). Remifentanil-PCA was discontinued during the second stage of labor when the parturient was actively pushing to deliver the baby, or in cases of pathological changes observed in the CTG, such as decreased variability, bradycardia, tachycardia, or late decelerations. According to institutional protocol, contraindications to remifentanil-PCA in labor included the parturient’s refusal, a history of opioid allergy, prior administration of parenteral opioids within the previous four hours, and the unavailability of one-to-one midwifery care.

For statistical analysis, we used IBM SPSS Statistics version 27.0 (IBM Corporation, Armonk, NY, USA). Continuous variables were described using either the median and interquartile range (25–75%) or the mean and standard deviation if the data were normally distributed. Normality of distribution was assessed using the Shapiro–Wilk test. Categorical variables were expressed as frequencies and proportions. Chi-square tests were employed to compare proportions, while the Kruskal–Wallis test was used for comparing continuous variables across different cervical dilation groups. A *p*-value of < 0.05 was considered statistically significant.

## 4. Results

**Figure 1:**
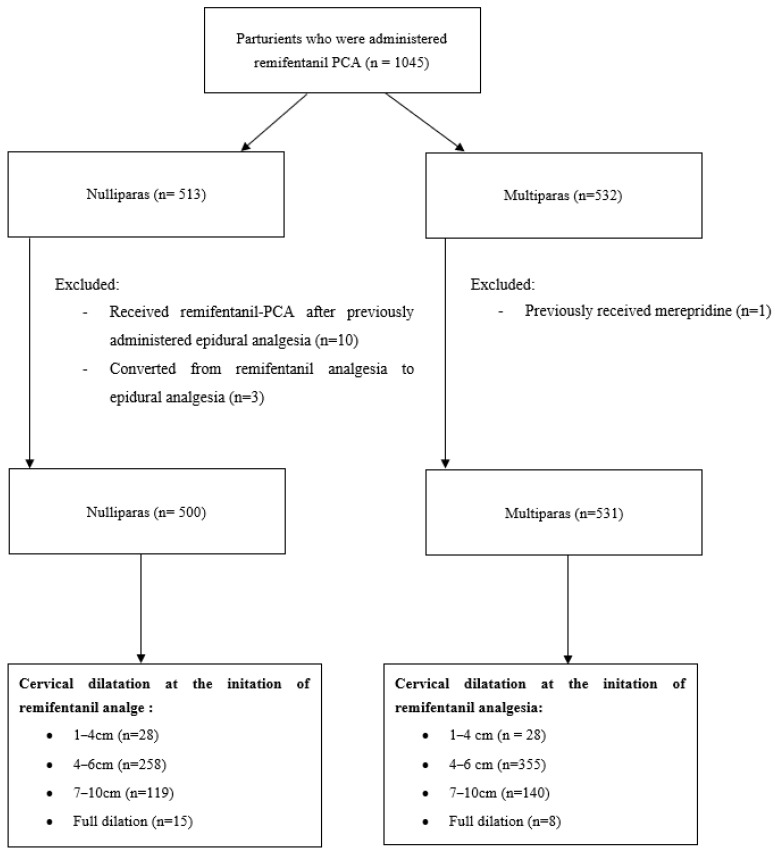
Flowchart.

During the study period, the records of 1045 laboring parturients were reviewed. They were categorized into four groups based on cervical dilation at the initiation of remifentanil analgesia: 1–3 cm, 4–6 cm, 7–9 cm, and 10 cm (full dilation). One multipara who had previously received pethidine, ten primiparas who received remifentanil-PCA after having epidural analgesia, and three primiparas who converted from remifentanil analgesia to epidural analgesia were excluded from the analysis (Figure 1).

Table 1 presents the demographic and obstetric/neonatal data categorized by parity (nulliparas vs. multiparas). No differences were observed between the study groups in terms of age, BMI, rate of labor inductions, the proportion of fetuses in the occipito-posterior position, proportion of parturients with blood loss > 500 mL, or neonatal outcomes. Nulliparas exhibited a higher gestational age (*p* = 0.021), longer labor duration (*p* < 0.001), and a greater proportion of cesarean sections (*p* < 0.001) and vacuum extractions (*p* = 0.002) compared to multiparas.

**Figure 2:**
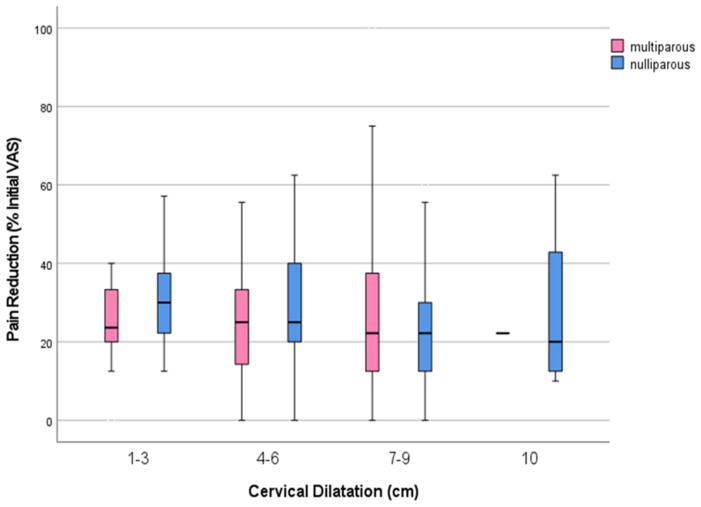
Reduction in pain by VAS according to cervical dilatation in Nulliparas and Multiparas. No statistically significant differences in pain reduction with respect to cervical dilation at the beginning of analgesia were observed in nulliparas and multiparas.

Table 2 presents analgesic data related to cervical dilation at the initiation of analgesia for both nulliparous and multiparous women. No significant differences were observed in the initial pain intensity (VAS_0_), pain intensity after one hour of remifentanil analgesia (VAS_1_), or in the extent of pain reduction (VAS_0_–VAS_1_) during the first hour of analgesia. However, among nulliparas, the lowest pain intensity (VAS_min_) varied with respect to cervical dilation, though its clinical relevance remains debatable (*p* < 0.026). Nevertheless, pain reduction was categorized as mild to moderate, regardless of the stage of labor at the initiation of analgesia and parity (Figure 2). Additionally, the concurrent use of nitrous oxide was significantly higher in nulliparas (227; 45.4%) compared to multiparas (175; 34%) (*p* < 0.001).

Data on satisfaction with remifentanil analgesia were collected from 370 nulliparas and 390 multiparas. Only two nulliparas (0.5%) and one multipara (0.3%) expressed dissatisfaction with labor analgesia. Furthermore, moderate and high satisfaction rates were significantly higher among multiparas compared to nulliparas (*p* < 0.001) (Table 3).

Desaturations below 94% were recorded in ten primiparas (0.02%) and seven multiparas (0.02%). Importantly, no severe complications, such as cardiac or respiratory arrest requiring artificial ventilation or intubation in either mothers or infants, were reported.

## 5. Discussion

This is the first study to examine the analgesic efficacy of remifentanil in relation to the phase of labor and parity. The results suggest that remifentanil’s effectiveness in reducing pain scores is not influenced by cervical dilation at the initiation of analgesia or by parity. Apart from its immediate availability and rapid analgesic effect, our study showed that remifentanil-PCA may be administered at any point during labor, enhancing the flexibility of its use in clinical practice. Such flexibility is particularly important in cases of rapidly progressing or advanced labor, especially when conventional epidural placement cannot be implemented immediately and its effect delayed [6,7].

Furthermore, our study demonstrated that remifentanil could effectively alleviate labor pain, specifically reducing the pain from severe (VAS 8–10) to moderate (VAS 4–7) in most cases. This finding is consistent with two meta-analyses comparing remifentanil-PCA with epidural analgesia [12,13]. Although remifentanil-PCA performed less favorably, a statistically significant decrease in 0–10 VAS scores was recorded within the first hour of remifentanil analgesia, ranging from −1 in the study by Volmanen et al. to −5 points in the study by Evron et al. (average reduction of −2.8 points) [12,13,14,15]. The same extent of pain reduction was noted in the study by Logtenberg et al., who reported a 2.8-point reduction in VAS scores [5]. Additionally, in the RESPITE study, where remifentanil-PCA was compared to intramuscular pethidine, the median VAS pain score was significantly reduced by 13.91 points on the 100 mm VAS scale in the remifentanil-PCA group compared to the pethidine group [16]. These findings are reflective of the capability of remifentanil-PCA to assist parturients in managing their discomfort during labor [17,18,19]. As a corollary, remifentanil-PCA may not be suitable for those seeking a completely pain-free labor experience [3,4,5,12]. Instead, it is more appropriate for individuals who prefer to avoid neuraxial analgesia or who wish to maintain some degree of control over their labor experience without achieving a complete pain reduction [5,17,18,19].

Despite the limited analgesic efficacy, the majority of parturients expressed high satisfaction with remifentanil-PCA, according to our scoring system. This satisfaction could be attributed to maternal expectations regarding pain relief among women who chose remifentanil-PCA as their preferred method. All parturients were counseled that they would not achieve complete pain relief with remifentanil-PCA. In contrast, women who requested neuraxial analgesia might have had higher expectations regarding analgesic effectiveness. Indeed, the subset of women who initially chose neuraxial analgesia but experienced incomplete relief often reported lower satisfaction [20]. Conversely, women who opted for remifentanil-PCA instead of neuraxial analgesia might value factors such as immediate availability, rapid onset of self-administered analgesia, the continuous presence of a midwife, and the euphoric and sedative effects of opioids on pain perception [3,5]. Of note, higher satisfaction among multiparas has also been found by Logtenberg et al., which may be attributed to shorter labor duration, fast availability and onset of analgesia with remifentanil-PCA, and a lower need for supplemental analgesia, such as nitrous oxide, compared to nulliparas [5].

In contrast to our previous report, where the oxygen desaturation rate was about 30%, this study revealed a relatively low rate of oxygen desaturation below 94% [3]. Besides the possibility of underreporting, this difference might be attributed to other factors, such as the use of incremental dosages of remifentanil without continuous infusion, mandatory monitoring, or the vigilance of the care providers, including the constant presence of the two anesthetic teams in the labor ward. However, it is noteworthy that no serious complications, such as the need for artificial ventilation, intubation, or cardiopulmonary resuscitation, were recorded among the mothers during this year-long study of more than 1000 parturients, indicating the relative safety of the use of remifentanil-PCA in our labor ward [3,21,22].

A Cochrane systematic review with meta-analysis comparing remifentanil-PCA to other parenteral methods for alleviating labor pain emphasized the need for data on the neonatal safety of remifentanil-PCA [23]. In our study, the percentage of Apgar scores below 7 five minutes after birth, instances of neonatal asphyxia, and admissions to the NICU was around 1.5%. Our results compared favorably with previous retrospective studies, where the need for neonatal resuscitation ranged from 0.08% to 1.48% in the absence of remifentanil-PCA [18,24,25]. Therefore, our findings could arguably support the relative safety of remifentanil-PCA for newborns. This safety could be attributed to the vigilance and close monitoring accorded with the mandatory cessation of the pump during the expulsion phase of labor.

Our study has limitations. First, as an observational study, we could not account for all of the potential confounding factors that might have influenced pain intensity and the efficacy of pain relief methods. Second, this study did not include a control group to compare labor and delivery outcomes. Nevertheless, the study comparing remifentanil-PCA and neuraxial analgesia in more than 10,000 deliveries showed that remifentanil-PCA was associated with lower cesarean section and operative delivery rates, with no differences in APGAR < 7 at 5 min, neonatal asphyxia, and NICU admission between the two analgesic techniques [21]. However, while correlations have been observed, they do not imply causation. Third, the research was conducted at a single center. While our tertiary center has the highest volume of remifentanil analgesia cases in our country and the surrounding area, our findings may not be generalizable to other settings.

## 6. Conclusions

Remifentanil-PCA demonstrates consistent effectiveness regardless of the stage of labor or parity, suggesting that it can be administered at any point during labor. Its availability for immediate analgesic effect further enhances the flexibility of its use in clinical practice. The results of this study could help inform mothers about the expected analgesic effects of remifentanil-PCA, aligning their expectations with the reality of their experience to help increase satisfaction with childbirth.

## Figures and Tables

**Table 1:** Demographic and perinatal data; data are presented as mean and standard deviation (±SD) or median and interquartile range [IQR].

	Nulliparas	Multiparas	*p*
Number of mothers (count)	500	531	
Age (years)	30 ± 5	32 ± 4	0.021 *
BMI (kg m^−2^)	28.6 ± 4.8	29.5 ± 4.9	0.485
Gestational age (weeks)	40 [28–43]	39 [32–43]	0.006 *
Induction of labor (number/percentage)	163 (33)	191 (36)	0.070
Duration of labor (min)	137 [7–694]	75 [6–374]	<0.001 *
Birth weight of newborn (g)	3345 ± 485	3509 ± 459	0.897
O/P position (number/percentage)	9 (1.8)	5 (0.9)	0.290
Blood loss after delivery > 500 mL (number/percentage)	41 (8.2)	37 (7)	
Cesarean section (number/percentage)	57 (11.4)	16 (3)	<0.001 *
Vacuum extraction (number/percentage)	18 (3.6)	4 (0.8)	0.002 *
Apgar score < 7 at 5 min (number/percentage)	4 (0.8)	0	0.057
Perinatal asphyxia pH in umbilical artery < 7 (number/percentage)	4 (0.8)	1 (0.2)	0.208
Neonatal admission to NICU (number/percentage)	5 (1)	1 (0.2)	0.116

Notes: BMI—body mass index, O/P position—occipito-posterior position, NICU—Neonatal Intensive Care Unit, * *p* < 0.05.

**Table 2:** Anesthetic data, pain intensity before the initiation of remifentanil analgesia (VAS_0_), pain intensity in the first hour of remifentanil analgesia (VAS_1_), pain intensity reduction in the first hour of remifentanil analgesia (VAS_0_–VAS_1_), the lowest pain intensity during remifentanil-PCA (VAS_min_), maximum dose of remifentanil during labor. Data are presented as median and interquartile range [IQR].

A. Nulliparas					
Cervical Dilatation Before the Initiation of Remifentanil Analgesia (cm)	1–3	4–6	7–10	Full Dilation	*p*
VAS_0_	9 [8–10]	9 [8–10]	9 [8–10]	8 [7–10]	0.402
VAS_1_	6 [5–7]	6 [5–8]	7 [5–8]	6 [4–8]	0.081
VAS_min_	6 [4–6]	5 [4–7]	6 [5–7]	5 [4–5]	0.026 *
Pain reduction (VAS_0_–VAS_1_)	3 [2–3]	2 [2–3]	2 [1–3]	2 [1–3]	0.141
Maximum dose of remifentanil used during labor (μg)	30 [20–40]	30 [10–40]	30 [20–40]	30 [20–30]	
**B.** **Multiparas**					
**Cervical Dilatation (cm)**	**1–3**	**4–6**	**7–10**	**Full Dilation**	***p***
VAS_0_	8 [7–9]	8 [8–9]	9 [8–10]	9 [9–9]	0.132
VAS_1_	6 [5–7]	6 [5–8]	7 [6–8]	6 [4–8]	0.487
VAS_min_	4 [4–5]	6 [4–7]	6 [5–7]	7 [7–7]	0.054
Pain reduction (VAS_0_–VAS_1_)	2 [1–2]	2 [0–6]	2 [0–6]	2 [2–2]	0.979
Maximum dose of remifentanil used during labor (μg)	30 [10–40]	30 [20–40]	30 [20–40]	30 [30–40]	

Notes: VAS—Visual analog scale (VAS; 0—no pain and 10—worst possible pain), * *p* < 0.05.

**Table 3:** Satisfaction with labor analgesia using remifentanil-PCA (* *p* < 0.05).

	Nulliparas	Multiparas	*p*
Satisfaction (number)	370	390	
Low (number/percentage)	2 (0.5)	1 (0.3)	0.53
Moderate (number/percentage)	35 (9.5)	96 (24)	<0.001 *
High (number/percentage)	333 (90)	293 (75)	<0.001 *

## Data Availability

The datasets used and/or analyzed during the current study are available from the corresponding author upon reasonable request.
